# Study of mitochondrial respiratory defects on reprogramming to human induced pluripotent stem cells

**DOI:** 10.18632/aging.100950

**Published:** 2016-04-26

**Authors:** Sandy S.C. Hung, Nicole J. Van Bergen, Stacey Jackson, Helena Liang, David A. Mackey, Damián Hernández, Shiang Y. Lim, Alex W. Hewitt, Ian Trounce, Alice Pébay, Raymond C.B. Wong

**Affiliations:** ^1^ Centre for Eye Research Australia, Royal Victorian Eye and Ear Hospital and Ophthalmology, Department of Surgery, The University of Melbourne, Melbourne, Australia; ^2^ Lions Eye Institute and University of Western Australia, Nedlands, Australia; ^3^ O'Brien Institute Department, St Vincent's Institute of Medical Research, Fitzroy, Australia; ^4^ School of Medicine, Menzies Institute for Medical Research, University of Tasmania, Tasmania, Australia

**Keywords:** cellular reprogramming, mitochondria, oxidative phosphorylation, induced pluripotent stem cells, Leber's hereditary optic neuropathy

## Abstract

Reprogramming of somatic cells into a pluripotent state is known to be accompanied by extensive restructuring of mitochondria and switch in metabolic requirements. Here we utilized Leber's hereditary optic neuropathy (LHON) as a mitochondrial disease model to study the effects of homoplasmic mtDNA mutations and subsequent oxidative phosphorylation (OXPHOS) defects in reprogramming. We obtained fibroblasts from a total of 6 LHON patients and control subjects, and showed a significant defect in complex I respiration in LHON fibroblasts by high-resolution respiratory analysis. Using episomal vector reprogramming, our results indicated that human induced pluripotent stem cell (hiPSC) generation is feasible in LHON fibroblasts. In particular, LHON-specific OXPHOS defects in fibroblasts only caused a mild reduction and did not significantly affect reprogramming efficiency, suggesting that hiPSC reprogramming can tolerate a certain degree of OXPHOS defects. Our results highlighted the induction of genes involved in mitochondrial biogenesis (*TFAM, NRF1*), mitochondrial fusion (*MFN1, MFN2*) and glycine production (*GCAT*) during reprogramming. However, LHON-associated OXPHOS defects did not alter the kinetics or expression levels of these genes during reprogramming. Together, our study provides new insights into the effects of mtDNA mutation and OXPHOS defects in reprogramming and genes associated with various aspects of mitochondrial biology.

## INTRODUCTION

Recent advances in reprogramming of somatic cells into human induced pluripotent stem cells (hiPSCs) provide tremendous potential for disease modeling, drug discovery and gene therapy development. Previous reports showed that the process of reprogramming can ‘rejuvenate’ somatic cells and erase many aging signatures, such as age-associated mitochondrial respiration defects [1], aging gene profile and nuclear-cytoplasmic compartmentalization [2].

Understanding the mechanisms involved in reprogramming may provide valuable insights into the aging process. Mitochondrial oxidative phosphorylation (OXPHOS), mediated by the electron transport chain to generate ATP, provides a major source of cellular energy. However, the impact of OXPHOS defects in the reprogramming process remains understudied.

Mitochondrial impairment is involved in many pathological diseases and in aging [3]. The mitochondrial DNA (mtDNA) mutator mice, which accumulate random mtDNA mutations in all tissues, develop severe OXPHOS defects, premature aging and exhibit shortened lifespan [4, 5]. Interestingly, mtDNA mutator mouse embryonic fibroblasts exhibit lower reprogramming efficiencies compared to wildtype, suggesting mitochondrial respiratory defects caused by mtDNA mutations have a strong impact on reprogramming [6]. Furthermore, using patient's fibroblasts with the heteroplasmic mtDNA disease Mitochondrial Encephalopathy Lactic Acidosis, and Stroke-like episodes (MELAS), Yokota *et al*. showed that reprogramming efficiency was significantly decreased in fibroblast clones with >90% mutant mtDNA (m.3243A>G) that caused severe mitochondrial respiratory dysfunction, whereas fibroblasts with less mutant mtDNA load have normal reprogramming efficiency [7]. These studies suggested a possible link between OXPHOS defects and reprogramming.

To avoid complications relating to mtDNA heteroplasmy, here we utilized a homoplasmic mtDNA disease model, Leber's Hereditary Optic Neuropathy (LHON), to study OXPHOS defects in reprogramming. LHON represents the most common mtDNA-linked disease, where homoplasmic mtDNA mutation(s) in complex I led to impairment in OXPHOS [8, 9]. Utilizing LHON as a homoplasmic mtDNA disease model, here we studied the effect of mitochondrial respiratory defects caused by different mtDNA mutations (m.11778G>C, m.14484T>C, m.4160T>C) on iPSC reprogramming. We show that mitochondrial respiratory defects caused by complex I mutations exert only a modest decrease in reprogramming efficiency, and does not alter expression of genes associated with mitochondrial biogenesis, fusion and glycine production. To our knowledge, this study is the first to utilize a homoplasmic mtDNA disease to study the role of mitochondria complex I defect during reprogramming to hiPSCs.

## RESULTS

### Presence of mtDNA mutations and complex I defects in LHON fibroblasts

**Figure 1:**
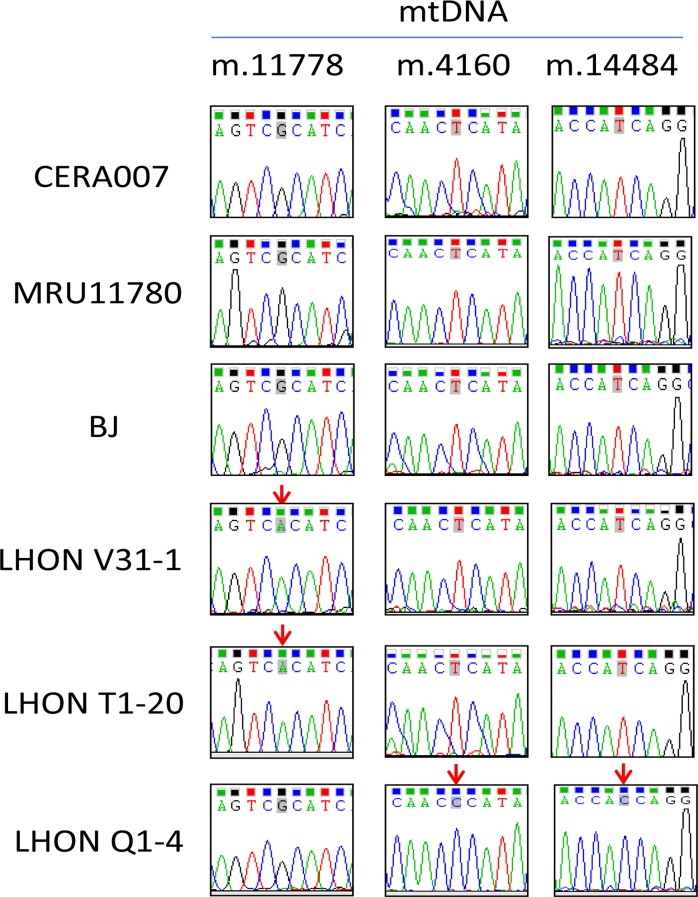
Genotyping of mtDNA mutation in patient fibroblasts Genotyping of mtDNA m.11778, m.4160 and m.14484 in patient-derived fibroblasts. Red arrows indicate the position of LHON mutation.

Table 1 summarizes the information of the patients involved in this study. We obtained skin fibroblasts from 3 control patients (CERA007, MRU11780 and BJ) and 3 LHON affected patients carrying different mtDNA mutations (LHON V31-1, LHON T1-20, LHON Q1-4). As shown in Figure 1, genotyping analysis revealed that LHON T1-20 and LHON V31-1 harboured the homoplasmic mtDNA mutation m.11778G>C in *MT-ND4* gene, representing the most common LHON mutation. LHON Q1-4 fibroblasts carried homoplasmic double mtDNA mutations m.4160T>C in *MT-ND1* gene and m.14484T>C in *MT-ND6* gene. This double mutation causes a more severe form of LHON known as the “LHON plus dystonia”, where patients exhibit optic nerve atrophy as well as a juvenile encephalopathy and peripheral neuropathy [10, 11]. On the other hand, the control fibroblasts (CERA007, MRU11780 and BJ) did not harbour mutation at any of the mtDNA positions sequenced (Figure 1).

**Table 1:** Information of patient samples used for reprogramming

Patient sample	Phenotype	mtDNA Genotype	Age (years)	Sex
CERA007	Healthy control	m.11778G m.4160T m.14484T	34	Male
MRU11780	Healthy control	m.11778G m.4160T m.14484T	90	Female
BJ	Healthy control	m.11778G m.4160T m.14484T	Neonatal	Male
LHON V31-1	LHON	m.11778G>C m.4160T m.14484T	18	Male
LHON T1-20	LHON	m.11778G>C m.4160T m.14484T	33	Male
LHON Q1-4	‘LHON plus’	m.11778G m.4160T>C m.14484T>C	30	Female

**Figure 2:**
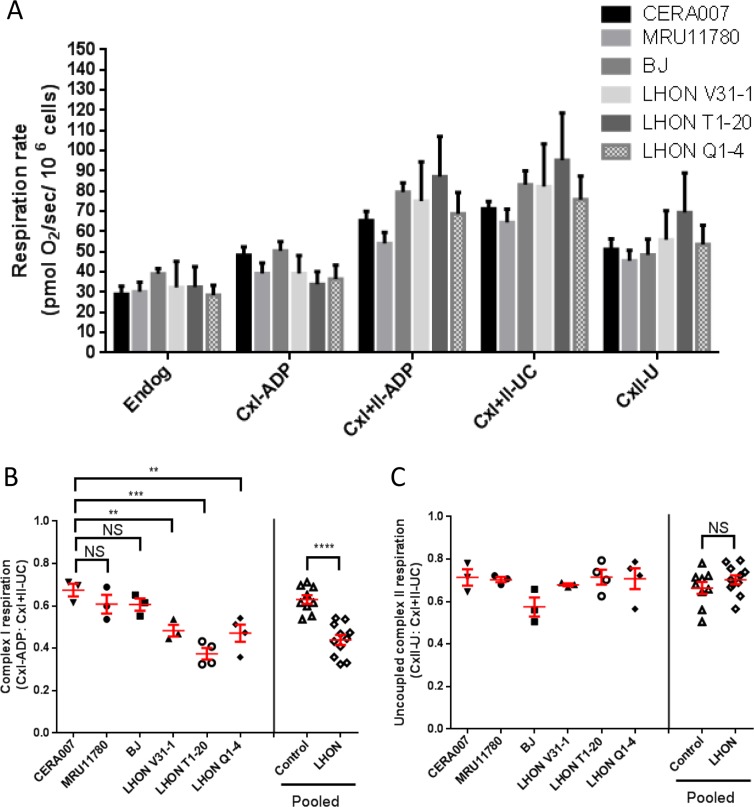
OXPHOS analysis of control and LHON fibroblasts (**A**) Oxygen consumption of control and LHON fibroblasts, showing the endogenous respiration rate (Endog), ADP-stimulated cell respiration with glutamate + malate (CxI-ADP) or with succinate (CxI+II-ADP), the uncoupled maximal respiration by addition of CCCP (CxI+II-UC) and the complex II respiration with uncoupled feedback by addition of rotenone (CxII-U). n = 3 for each patient fibroblast. Error bars represent SEM. (**B**) Complex I respiration of individual fibroblast samples and pooled data, normalized to uncoupled maximal respiration (CxI-ADP/CxI+II-UC). (**C**) Uncoupled complex II respiration (CxII-U/CxI+II-UC) in control (CERA007, MRU11780, BJ) and LHON fibroblasts (LHON V31-1, LHON T1-20, LHON Q1-4). **** = p<0.0001, *** = p<0.001, ** = p<0.01, * = p<0.05, ns = not significant.

Next, we performed high resolution respirometry to assess the mitochondrial respiration in control and LHON fibroblasts. Oxygen consumption rate at the endogenous level and the maximal uncoupled rate (after addition of carbonyl cyanide m-chlorophenyl hydrazone, CCCP) did not change significantly between control and LHON fibroblasts (Figure 2A). Also, the non-mitochondrial residual oxygen consumption rate, after addition of rotenone and antimycin A, was less than 1% of the maximal uncoupled rate (data not shown). To account for any differences in mitochondrial density per cell, ADP-stimulated complex I respiration can be normalized to the maximal uncoupled rate [12]. Following normalization to differences in mitochondrial density, our results showed that complex I activities were significantly lower in all three LHON fibroblasts (LHON V31-1, LHON T1-20, LHON Q1-4) compared to controls (Figure 2B). In contrast, there was no statistically significant difference in the uncoupled complex II respiration in control and LHON fibroblasts (Figure 2C). Together, our results confirmed that LHON mutations impaired mitochondrial complex I respiration in fibroblasts, a result consistent with those previously observed in other cell types from LHON patients, such as lymphoblasts [13, 14].

### Reprogramming of LHON fibroblasts

Using the control and LHON fibroblasts, we generated iPSCs in a feeder-free system by overexpression of six reprogramming factors OCT4, SOX2, KLF4, L-MYC, LIN28 and shRNA for p53 [15, 16]. On day 28 post-reprogramming, we assessed the reprogramming efficiency of the fibroblasts by quantification of colonies that express TRA-1-60, a marker previously used to identify fully reprogrammed hiPSCs [17].

**Figure 3:**
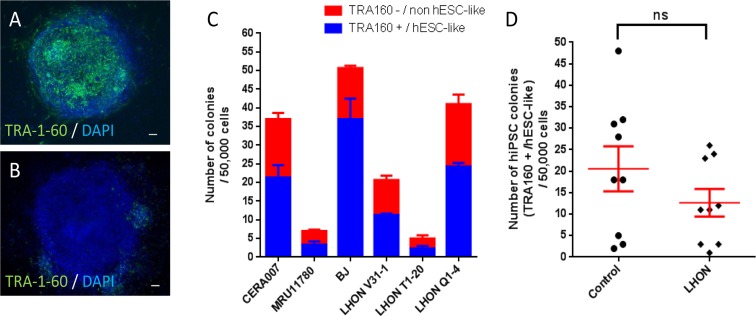
Reprogramming of control and LHON fibroblasts using feeder-free system Representative pictures of (**A**) TRA-1-60 positive colony and (**B**) TRA-1-60 negative colony. (**C**) Quantification of hiPSC colonies generated from control and LHON fibroblasts. Blue bar indicates *bona fide* hiPSC colonies, as defined by TRA-1-60 expression and hESC-like morphology, per 50,000 reprogrammed cells. Red bar indicates partially reprogrammed colonies as defined by absence of TRA-1-60 expression and/or resemble non-hESC-like morphology, per 50,000 reprogrammed cells. n=3, error bars represent SEM. (**D**) Pooled results of hiPSC colony number generated from control and LHON fibroblasts. ns = not significant, as indicated by unpaired t-test.

Figures 3A and 3B showed representative pictures of a fully reprogrammed hiPSC colony (TRA-1-60 positive) and a partially reprogrammed colony (TRA-1-60 negative). As shown in Figure 3C, we observed the highest number of TRA-1-60 positive colonies in BJ (37 ± 6), followed by 21 ± 3 and 24 ± 1 colonies in CERA007 and LHON Q1-4 respectively, and low colony numbers in LHON V31-1, MRU11780 and LHON T1-20 (11 ± 0, 3 ± 1 and 2 ± 1 respectively). In addition, we observed that across all cell lines ~30-50% of the colonies were not fully reprogrammed, as indicated by lack of TRA-1-60 expression or non-human embryonic stem cell (hESC)-like morphology (Figure 3C). Importantly, overall we observed a modest decrease in hiPSC colonies generated from LHON fibroblasts compared to control, (13 ± 3 versus 21 ± 5 respectively), however this difference in reprogramming efficiency was not statistically significant (*p* = 0.2158, Figure 3D). Thus, our results indicated that mitochondrial respiratory defects did not significantly affect the hiPSC reprogramming process.

### Characterization of derived hiPSCs

**Figure 4:**
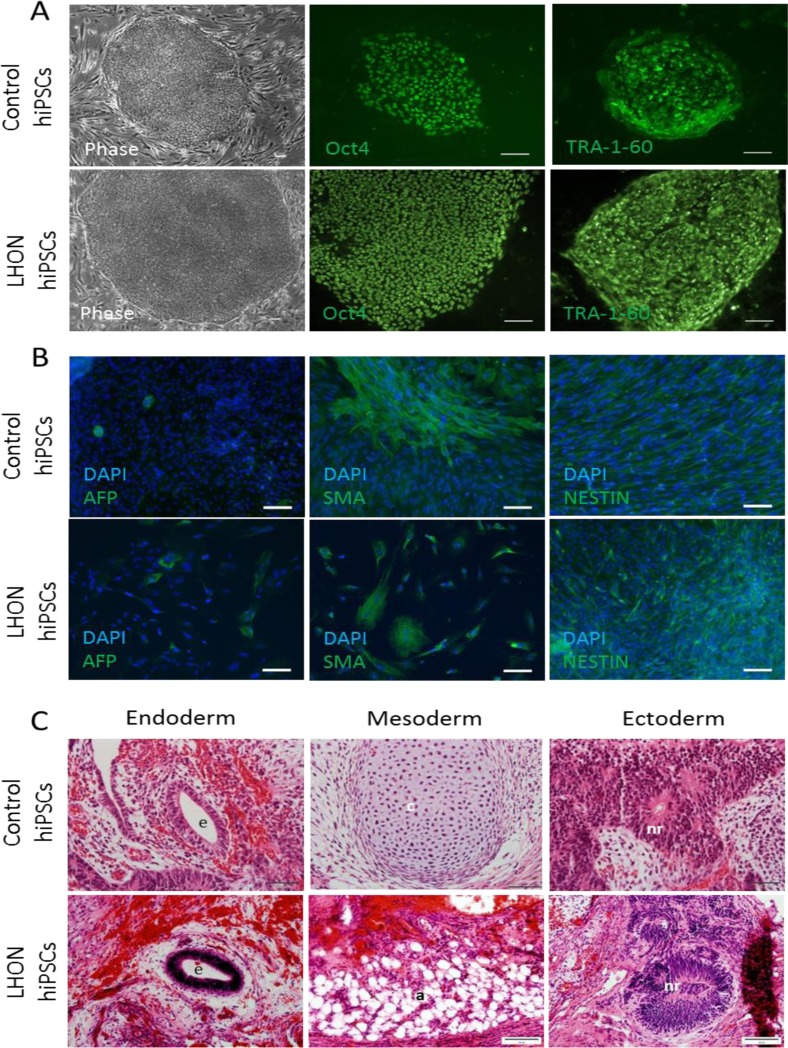
Characterization of the derived hiPSCs (**A**) Cell morphology and immunocytochemistry analysis of pluripotent markers OCT4 and TRA-1-60 in a representative control hiPSC line (MRU11780) and LHON hiPSC line (LHON Q1-4). Scale bar = 100μm. (**B**) *In vitro* embryoid body differentiation of hiPSC into cell representative of the endoderm (AFP), mesoderm (SMA) and ectoderm (NESTIN). Scale bar = 100μm. (**C**) hiPSCs form teratoma containing endodermal cells (e, gut-like epithelium), mesodermal cells (c, cartilaginous structure; a, adipose tissue) and ectodermal cells (nr, neural rosette). Scale bar = 50μm.

To ensure that the TRA-1-60 positive colonies are fully reprogrammed, we isolated the colonies by manual dissection to establish clonal hiPSC lines and performed a detailed characterization of a representative clonal hiPSC line from control (MRU11780) and LHON patient (LHON Q1-4). As illustrated in Figure 4A, the derived hiPSC colonies exhibited morphology similar to hESCs with a defined colony boundary and high cytoplasmic to nucleus ratio, as well as strong expression of the pluripotent markers OCT4 and TRA-1-60 by immunocytochemistry.

Upon spontaneous differentiation by embryoid body formation, the derived hiPSCs were able to differentiate into the three germ layers, including endodermal cells (AFP positive expression, Figure 4B), mesoderm cells (SMA positive expression, Figure 4B) and ectodermal cells (NESTIN positive expression Figure 4B). Furthermore, we transplanted the derived hiPSCs into nude rats using a vascularized chamber system described previously [16, 18]. After 4 weeks post-transplantation, we observed teratoma formation. As shown in Figure 4C, the resultant teratoma consisted of cells representative of endoderm (gut-like epithelium), mesoderm (cartilaginous structure or adipose tissues) and ectoderm (neural rosettes). Notably, both normal (MRU11780) and LHON hiPSCs (LHON Q1-4) were able to consistently form teratoma in all attempts (3 clonal hiPSC lines per patient, 2 transplantations into rat per clonal line). In this regard, we did not observe any notable differences in the quality of hiPSCs derived from normal and LHON fibroblasts. Together, these results demonstrated that the derived hiPSCs retained the potential to differentiate into the 3 germ layers both *in vitro* and *in vivo*, providing strong support that the derived hiPSCs from control and LHON patients were *bona fide*.

### Regulation of mitochondrial biogenesis, fusion and glycine production during reprogramming

**Figure 5:**
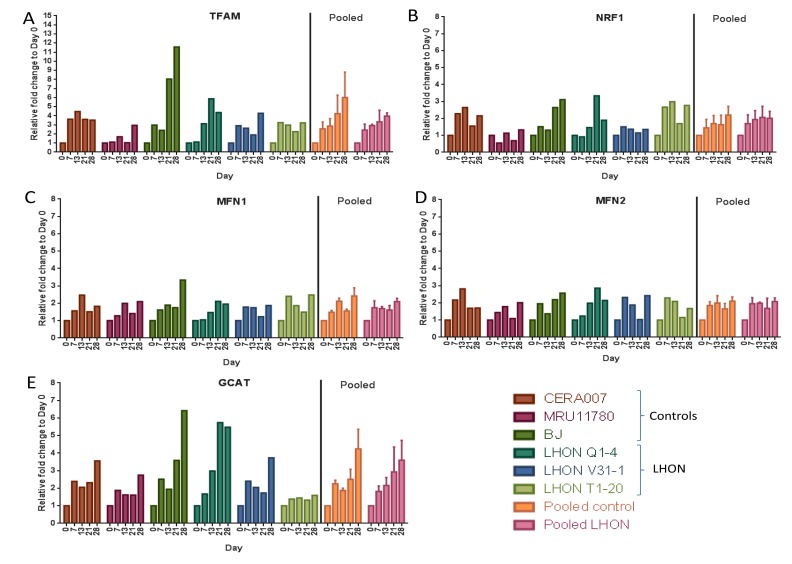
Expression of mitochondrial regulatory genes during reprogramming qPCR analysis of the expression of (A) *TFAM*, (B) *NRF1*, (C) *MRN1*, (D) *MFN2* and (E) *GCAT* at various time points during reprogramming to hiPSCs (day 0, 7, 13, 21, 28). Error bars represent SEM for pooled data of control (CERA007, MRU11780, BJ) or LHON (LHON Q1-4, LHON V31-1, LHON T1-20).

Finally, we tested if OXPHOS defects in LHON patient fibroblasts could alter regulation of multiple mitochondrial processes during reprogramming. Using qPCR, we assessed the expression of nuclear genes regulating mitochondrial biogenesis (*TFAM, NRF1*), mitochondrial fusion (*MFN1, MFN2*) and mitochondrial glycine production (*GCAT*) at various time points (days 7, 13, 21, 28) during reprogramming of control and LHON fibroblasts. Our results showed that genes involved in mitochondrial biogenesis, *TFAM* and *NRF1*, were both gradually upregulated during reprogramming, with a ~4 to 6-fold increase in *TFAM* expression (Figure 5A) and ~2-fold increase in *NRF1* expression by day 28 in pooled control or LHON samples (Figure 5B). On the other hand, *MFN1* and *MFN2*, both previously shown to be involved in regulating mitochondrial fusion, were modestly upregulated during reprogramming, reaching just ~2 fold increase by day 28 (Figure 5C and 5D). Finally, *GCAT* expression that was linked to mitochondrial glycine production was strongly upregulated during reprogramming in a gradual manner, reaching ~5-fold increase by day 28 (Figure 5E). Together, our results indicated an increase in expression of key genes regulating mitochondrial biogenesis and glycine production upon induction of pluripotency. However, overall we did not observe any significant differences in pooled data of LHON fibroblasts compared to controls, suggesting that the LHON-specific OXPHOS defects did not alter expression of genes regulating mitochondrial bio-genesis, fusion and glycine production.

## DISCUSSION

The roles of mitochondria and metabolism in the regulation of pluripotency and differentiation have been subjected to intense research in the past [19]. While pluripotent stem cells mainly utilize anaerobic glyco-lysis for energy production, somatic differentiated cells primarily rely on aerobic metabolism by OXPHOS for energy production [20]. Previous research has shed light onto the requirement for metabolic transition during reprogramming, from OXPHOS dependency in the somatic cells to ATP-generating glycolysis in iPSCs [21]. Here we focused on the role of mitochondrial OXPHOS in the induction of pluripotency during reprogramming. hiPSCs have been successfully generated from a range of mitochondrial diseases caused by mtDNA mutations, including MELAS [22-24], Leigh syndrome [25], Pearson marrow pancreas syndrome [26] and diabetes mellitus [27]. However, issues with mtDNA heteroplasmy make it difficult to interpret the impact of mtDNA mutation and subsequent OXPHOS defects in reprogramming.

The mtDNA mutational load in the cells often directly affects the outcome of the heteroplasmic mitochondrial disease. In the case of MELAS, fibroblasts carrying >90% of mutated mtDNA m.3243A>G showed significantly decreased reprogramming efficiency [7]. To avoid the complication with mtDNA heteroplasmy, this study utilized patient-derived LHON fibroblasts carrying homoplasmic mtDNA mutations. We observed a significant defect in complex I respiration in all LHON fibroblasts. Interestingly, the double mutations at mtDNA m.4160T>C + m.14484T>C, that caused the more severe ‘LHON plus’ phenotype, resulted in a similar degree of complex I defects compared to the most common LHON mutation at mtDNA m.11778G>C. Despite these OXPHOS defects in LHON fibroblasts, our results indicated that reprogramming to hiPSCs was still amenable. Using an integration-free, feeder-free reprogramming system, we demonstrated that the overall OXPHOS defects caused by LHON exert only a mild, but not statistically significant, impairment in hiPSC reprogramming. Our results supported the notion that the process of reprogramming can tolerate a certain degree of OXPHOS defects caused by complex I dysfunction. It is possible that OXPHOS defects may have different impacts on reprogramming in a different starting cell type. For instance, mouse embryonic fibroblast from mtDNA mutator mice exhibit a significantly decreased reprogramming efficiency to generate iPSCs [6], however the hematopoietic progenitor cells seem to exhibit a similar reprogramming efficiency compared to controls [28]. Future research to assess reprogramming of different somatic cell types with OXPHOS defects will help clarify this issue. Also, a potential limitation of this study is that the control and LHON patients are from a range of different ages and have different genetic backgrounds. This should be taken into consideration when interpreting this study. Nevertheless, regardless of differences in age and genetic background, our results clearly indicated that the LHON mtDNA mutation caused significant complex I defects in fibroblasts, and this in turn did not result in a detrimental effect on reprogramming efficiency.

We also examined other aspects of mitochondria regulation during the process of reprogramming. For instance, mitochondrial fusion has been implicated as a barrier to reprogramming. Depletion of Mfn1/2 has been shown to impair mitochondrial fusion and promote reprogramming previously [29]. In support of this, a previous report by Vazquez-Martin *et al*. demonstrated that reprogramming of mouse embryonic fibroblasts was significantly impaired following pharmacological inhibition of the GTPase dynamin DRP1, which selectively inhibits mitochondrial fission and promotes mitochondrial fusion [30]. This is consistent with our results, where reprogramming is accompany by only modest changes in *MFN1* and *MFN2* expression. Moreover, mitophagy has also been implicated in reducing mitochondria number in differentiated cells and contributes to mitochondrial remodeling events that occurred during reprogramming [30, 31]. On the other hand, the role of mitochondrial biogenesis is less clear during reprogramming. NRF1 is an upstream regulator of TFAM, a nuclear factor that is critical for mtDNA transcription and replication [32, 33], where disruption of this pathway resulted in decreased mtDNA copy number [34]. In terms of reprogramming, previous research have shown that fibroblasts exhibit higher mtDNA contents compared to hiPSCs [20]. Unexpectedly, our results showed that expression of *TFAM* and to a lesser extent *NRF1* are both upregulated during reprogramming, suggesting that the re-programmed cells are still capable of mtDNA transcription and replication through the NRF1/TFAM pathway. It is worth noting that induction of nuclear factors involved in mitochondria biogenesis, such as TFAM, has been described in response to mtDNA depletion [35]. Further studies into the mechanisms regulating mitochondrial biogenesis will provide better insight into its role during reprogramming. In addition, Masotti *et al*. showed that prolonged culture of hiPSC can alter the levels of mitochondrial biogenesis, leading to increased expression of *TFAM* and *NRF1* and increased level of mtDNA content [36]. This further suggests a dynamic change in mitochondria biogenesis not only during reprogramming to generate hiPSCs but also in prolonged maintenance of hiPSCs.

Previous studies have established a link between cellular age and hiPSC reprogramming. In a large scale study of 298 patient samples, Paull *et al*. showed that increasing age impeded the efficiency of reprogramming to hiPSCs [37]. In our set of patient samples, we observed a loose correlation where increasing patient age negatively influence reprogramming efficiency (Pearson correlation value = −0.6733). When comparing elderly fibroblasts versus young fibroblasts, an elegant study by Hashizume *et al*. showed that age-associated mitochondrial OXPHOS defects were partly caused by downregulation of *GCAT* gene, which is involved in mitochondrial glycine production [1]. Interestingly, the reprogramming process induced *GCAT* expression and effectively rescued the age-associated OXPHOS defects in elderly fibroblasts [1]. Here we provide new insight that *GCAT* expression is upregulated during re-programming of human fibroblasts as early as 7 days. This induction of *GCAT* expression seems to be independent of the age of the patient fibroblasts, as we observed in fibroblasts ranging from neonatal to 90 years old. Importantly, our results suggested that LHON-specific mtDNA mutations and OXPHOS defects did not alter genes associated with mitochondrial glycine production (*GCAT*), mitochondrial biogenesis (*NRF1, TFAM*) and fusion (*MFN1, MFN2*) during reprogramming.

Finally, future research to unravel the role of mitochondrial oxidative stress during reprogramming would be interesting. In particular, mitochondrial reactive oxygen species (ROS) have been shown to play a key role in modulating the mitochondrial adaptive response (also known as mitohormesis) and contributing to the life-extending effects of calorie restriction [38, 39]. Detailed analysis of the role of ROS and mitohormesis during reprogramming to iPSCs may explain how the re-programming process can rejuvenate age-associated mitochondrial respiration defects. In this regards, previous work in cybrids demonstrated that LHON mtDNA mutations increased ROS levels [40, 41]. How-ever, as this has not been comprehensively demonstrated in LHON fibroblasts, we cannot exclude the possible role of ROS in reprogramming of fibroblasts to hiPSCs.

In conclusion, this study demonstrated that the reprogramming process can tolerate a certain degree of OXPHOS defects without significant impact on efficiency of hiPSC generation, nor gene regulation of mitochondrial glycine production, biogenesis and fusion. Our results provided further understanding of the extensive mitochondrial restructuring events during reprogramming of somatic cells to pluripotent stem cells. Future studies to investigate hiPSC generation from other homoplasmic mitochondrial diseases will provide insights into the effects of impaired mito-chondrial respiration in hiPSC reprogramming.

## METHODS

### Ethical approval

Experimental procedures involving animals were approved by the Animal Ethics Committee of St. Vincent's Hospital (AEC 002/14; Victoria, Australia) and were conducted in accordance with the Australian National Health and Medical Research Council guidelines for the care and maintenance of animals. Experiments involving human cell lines and collection of patient samples were approved by the Human Research Ethics committees of the Royal Victorian Eye and Ear Hospital (11/1031H, 13/1151H-004) and University of Melbourne (0605017, 0829937) and carried out in accordance with the approved guidelines.

### Cell culture

Human fibroblasts were cultured in DMEM medium supplemented with 10% fetal calf serum, 1 x L-glutamine, 0.1 mM non-essential amino acids and 0.5× penicillin/streptomycin (all from Invitrogen). hiPSCs were cultured on mitotically inactivated mouse embryonic fibroblasts feeders in the presence of DMEM/F-12 medium containing 1 x GlutaMAX, 20% knockout serum replacement, 10 ng/ml basic fibroblast growth factor, 0.1 mM non-essential amino acids, 100 μM β-mercaptoethanol and 0.5× penicillin/streptomycin (all from Invitrogen).

### Reprogramming to hiPSCs

hiPSCs were generated from skin fibroblasts from patients using episomal vectors in a feeder-free system. Briefly, 50,000 skin fibroblasts were nucleofected with episomal vectors expressing OCT4, SOX2, KLF4, L-MYC, LIN28 and shRNA against p53 [15, 16, 42]. On day 0, the nucleofected fibroblasts were plated on vitronectin XF-coated 6-well plates (Stem Cell Technologies) in fibroblast media. On day 2, the cells were switched to E7 media (Stem Cell Technologies). On ~day 30, reprogrammed colonies resembling hESC morphology were manually dissected to establish clonal cell lines for expansion and characterization. Note that the characterization of a particular clonal line (CERA007c6) from the control patient CERA007 has been described in our previous publication [42].

### Quantification of hiPSC reprogramming

Day 28 reprogrammed cells were fixed with 4% para-formaldehyde and immunostained with TRA-1-60 antibodies (5μg/ml, Millipore) followed by the appropriate Alexa-Fluor 488 secondary antibodies. The samples were viewed under a fluorescent microscope (Nikon Eclipse TE-2000U) and manual counting was performed to quantify the number of hiPSC colonies per well, as defined by TRA-1-60 positive colonies that resemble hESC-like morphology.

### High resolution respirometry analysis

The Oroboros Oxygraph 2K (Oroboros Instruments) was used to measure cellular respiration in LHON and control fibroblasts. Briefly, 500,000 cells per chamber were permeabilized with digitonin to allow access of OXPHOS substrates as described previously [43]. Respiration states of the samples were measured including the endogenous respiration rate, maximal complex-I ADP stimulated respiration (with ADP, glutamate and malate) then maximal complex-I+II respiration (after the addition of succinate), followed by the uncoupled maximal respiration (after the addition of CCCP). Residual oxygen consumption was measured after the addition of antimycin A and rotenone.

### Quantitative PCR

RNA samples were harvested from fibroblasts at various time points during reprogramming using the RNeasy kit and treated with DNase I (Qiagen). cDNA synthesis was performed using the High capacity cDNA reverse transcription kit (Applied Biosystems). Taqman assays were performed using probes for *TFAM* (Hs01082775_m1), *NRF1* (Hs01031046_m1), *MFN1* (Hs00966851_m1), *MFN2* (Hs00208382_m1), *GCAT* (Hs00606568_gH) and the housekeeping gene *β-ACTIN* (Hs99999903_m1) following standard procedure (all from Applied Biosystems). Samples were processed using the Step One plus real time PCR system (Applied Biosystems) and analysed by ΔΔCt method.

### Immunocytochemistry

Standard immunocytochemistry procedure was performed as previously described [44]. Primary antibodies used were TRA-1-60 (5μg/ml, Millipore), OCT4 (5μg/ml, Santa Cruz Biotechnology), VIMENTIN (2μg/ml, Calbiochem, #IF01), α-smooth muscle actin (10μg/ml, SMA, R&D Systems), NESTIN (10μg/ml, Abcam) and alpha-fetoprotein (AFP, 10μg/ml, Millipore). The samples were subsequently immunostained with the appropriate Alexa Fluor-488 antibodies, followed by DAPI nuclear counterstain. Samples were imaged using a Nikon Eclipse TE2000 inverted microscope or Zeiss Axio Imager Microscope.

### *In vitro* and *in vivo* differentiation of hiPSCs

For *in vitro* differentiation, embryoid bodies were formed in suspension culture for 11 days, and subsequently plated on gelatinized dishes to differentiate further for 18 days. Cells representative of the three germ layers were detected by marker expression using immunocyto-chemistry (SMA, NESTIN and AFP). For *in vivo* differentiation, teratoma were formed by transplanting ~2×10^6^ hiPSCs into a vascularized tissue engineering chamber in immune-deficient rats [16, 18]. Teratoma constructs were harvested 4 weeks after implantation for histological analysis.

### Statistical analysis

Statistical analysis was performed using Prism 3.0 program (GraphPad Software). One-way ANOVA analysis followed by Dunnett's test was performed for comparison with multiple groups. The student's t-test was performed for comparison between two groups. *p* < 0.05 was used to establish statistical significances.

## SUPPLEMENTARY FIGURE


