# Supplementary material for: Study of mitochondrial respiratory defects on reprogramming to human induced pluripotent stem cells
Source: Aging (Albany NY). 2016 Apr 26;8(5):945–56. doi: 10.18632/aging.100950 (PMC4931846; doi:10.18632/aging.100950)
Supplement: Supplementary file 1 [file aging-08-0945-s001.pdf]

SUPPLEMENTAL FIGURE

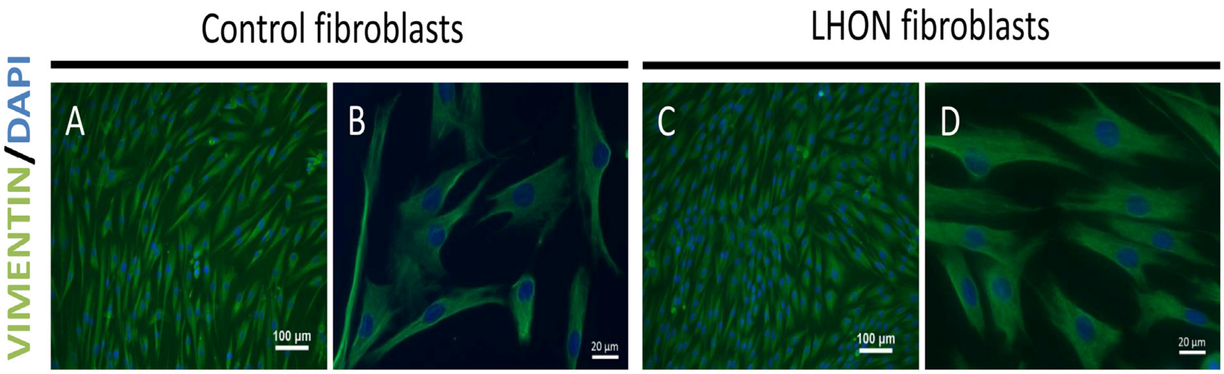

Supplemental Figure 1. Confirmation of purity of fibroblast cultures. Immunocytochemistry analysis of fibroblast marker VIMENTIN in (**A**-**B**) control (MRU11780) and (**C**-**D**) LHON fibroblasts (LHON Q1-4). Images are taken using (**A**,**C**) 10× and B,D) 40× objective lenses.
